# The Perioperative Quality Improvement Programme (PQIP patient study): protocol for a UK multicentre, prospective cohort study to measure quality of care and outcomes after major surgery

**DOI:** 10.1186/s13741-022-00262-3

**Published:** 2022-08-09

**Authors:** S. Ramani Moonesinghe, Dermot McGuckin, Peter Martin, James Bedford, Duncan Wagstaff, David Gilhooly, Cristel Santos, Jonathan Wilson, Jenny Dorey, Irene Leeman, Helena Smith, Cecilia Vindrola-Padros, Kylie Edwards, Georgina Singleton, Michael Swart, Rachel Baumber, Arun Sahni, Samantha Warnakulasuriya, Ravi Vohra, Helen Ellicott, Anne-Marie Bougeard, Maria Chazapis, Aleksandra Ignacka, Martin Cripps, Alexandra Brent, Sharon Drake, James Goodwin, Dorian Martinez, Karen Williams, Pritam Singh, Matthew Bedford, Abigail E. Vallance, Katie Samuel, Jose Lourtie, Dominic Olive, Christine Taylor, Olga Tucker, Giuseppe Aresu, Andrew Swift, Naomi Fulop, Mike Grocott, for the PQIP Project team and Clinical Reference Group, Ramani Moonesinghe, Giuseppe Aresu, Rachel Baumber, James Bedford, Matthew Bedford, Alexandra Brent, Maria Chazapis, Jake Comish, Martin Cripps, Jenny Dorey, Sharon Drake, Naomi Fulop, David Gilhooly, James Goodwin, Mike Grocott, Irene Leeman, Peter Martin, Claire McCann, Dermot McGuckin, Leila Nasser, Arun Sahni, Pritam Singh, Helena Smith, Chris Snowden, John Stones, Mike Swart, Olga Tucker, Abigail Vallance, Cecilia Vindrola, Ravi Vohra, Duncan Wagstaff, Karen Wiliams, Jonathan Wilson, John Abercrombie, Suhail Anwar, Anna Bachelor, Stephen Brett, Tom Clark, Graham Cooper, Anna Crossley, Jugdeep Dhesi, Marie Digner, Elspeth Evans, Mark Hamilton, Robert Hill, Zoe Huish, Ravi Mahajan, Dave Murray, Monty Mythen, Jonathan McGhie, John McGrath, Samantha Shinde, Mark Speakman, Andrew Swift, Emma Vaux

**Affiliations:** 1 Centre for Perioperative Medicine, Research Department for Targeted Intervention, UCL, London, UK; 2 Health Services Research Centre, Royal College of Anaesthetists, London, UK; 3 Department of Anaesthesia and Perioperative Medicine, University College London Hospitals NHS Foundation Trust, London, UK; 4 Department for Applied Health Research, UCL, London, UK; 5 Department of Anaesthesia, York Teaching Hospitals NHS Foundation Trust, York, UK; 6 Royal College of Anaesthetists, London, UK; 7 Department of Anaesthesia, Royal National Orthopaedic Hospital, Stanmore, UK; 8 Department of Anaesthesia, Torbay Hospital, Torquay, UK; 9 Department of Upper GI Surgery, Nottingham University Hospitals, Nottingham, UK; 10 NetSolving Limited, Croydon, UK; 11 Trent Oesophago-Gastric Unit, Nottingham University Hospitals NHS Trust, Nottingham, UK; 12 Department of Colorectal Surgery, Birmingham Heartlands Hospital, Birmingham, UK; 13 Bristol Medical School, University of Bristol, Bristol, UK; 14 Department of Anaesthesia, North Bristol NHS Foundation Trust , Bristol, UK; 15 Department of Upper Gastrointestinal Surgery, Heartlands Hospital, Birmingham, UK; 16 Department of Cardiothoracic Surgery, Papworth Hospital, Cambridge, UK; 17 Aintree Hospital, Liverpool, UK; 18 Division of Critical Care, University of Southampton, Southampton, UK

## Abstract

**Introduction:**

Major surgery accounts for a substantial proportion of health service activity, due not only to the primary procedure, but the longer-term health implications of poor short-term outcome. Data from small studies or from outside the UK indicate that rates of complications and failure to rescue vary between hospitals, as does compliance with best practice processes. Within the UK, there is currently no system for monitoring postoperative complications (other than short-term mortality) in major non-cardiac surgery. Further, there is variation between national audit programmes, in the emphasis placed on quality assurance versus quality improvement, and therefore the principles of measurement and reporting which are used to design such programmes.

**Methods and analysis:**

The PQIP patient study is a multi-centre prospective cohort study which recruits patients undergoing major surgery. Patient provide informed consent and contribute baseline and outcome data from their perspective using a suite of patient-reported outcome tools. Research and clinical staff complete data on patient risk factors and outcomes in-hospital, including two measures of complications. Longer-term outcome data are collected through patient feedback and linkage to national administrative datasets (mortality and readmissions). As well as providing a uniquely granular dataset for research, PQIP provides feedback to participating sites on their compliance with evidence-based processes and their patients’ outcomes, with the aim of supporting local quality improvement.

**Ethics and dissemination:**

Ethical approval has been granted by the Health Research Authority in the UK. Dissemination of interim findings (non-inferential) will form a part of the improvement methodology and will be provided to participating centres at regular intervals, including near-real time feedback of key process measures. Inferential analyses will be published in the peer-reviewed literature, supported by a comprehensive multi-modal communications strategy including to patients, policy makers and academic audiences as well as clinicians.

## Strengths and limitations of this study

### Strengths

- The largest observational study of patients undergoing major surgery in the UK National Health Service.
- The first national effort to measure and report risk-adjusted complications, patient-reported outcome and mortality rates for patients undergoing major surgery in the UK.

### Limitations

- The requirement for patient consent means that the sample may be biased. While this is of less importance for process measures which will be used to evaluate care quality, it is relevant when considering outcomes. We will mitigate this risk through comparison of the PQIP population to the total sample of patients undergoing PQIP included procedures using Hospital Episode Statistics (HES) data. This will enable us to undertake sensitivity analyses which will demonstrate what (if any) biases there are in our sampling.
- Similarly, our ambition to enable as many hospitals as possible to participate irrespective of local resourcing, means that some hospitals will attempt to recruit all patients who are potentially eligible, and some only a sample. We will again try to mitigate this risk through encouraging hospitals to recruit a random sample of patients and will compare the PQIP sample against the HES-defined population at local level as well.

## Introduction

Approximately 1.5 million major surgical procedures are carried out in the UK National Health Service per year at a cost of around £5 billion (Abbott et al. 2017); globally, surgical caseload substantially exceeds 300 million procedures per annum (Weiser et al. 2015). International estimates of death within 30 days of planned (elective or expedited) surgery vary, but it is acknowledged to be relatively uncommon in high income nations (around 0.6% to 4% depending on the study design, health system and population characteristics) (Abbott et al. 2017; Pearse et al. 2012; Ghaferi et al. 2009). However, there are several reasons why these low estimates of short-term mortality may provide false reassurance about the quality of perioperative care and the potential health and economic burden on patients and society. First, the incidence of major or prolonged postoperative morbidity is at least ten times higher than short-term mortality (Moonesinghe et al. 2014). This is important, because major morbidity contributes to increased length of hospital stay and associated increased cost of healthcare. Second, US data have shown wide variation in risk-adjusted morbidity and failure-to-rescue rates between the sample of healthcare providers who voluntarily participate in the American College of Surgeons’ National Surgical Quality Improvement Program (ACS-NSQIP), suggesting that at least some of these poor short-term outcomes could be avoidable (Ghaferi et al. 2009; Ghaferi et al. 2009). However, the lack of a unified national system for measuring complications across different types of major surgery in the NHS means that it is currently not possible to ascertain if the UK has the same problem. Third, a consistent finding across different procedures, populations and healthcare systems is the independent association between short-term postoperative complications and reduced longer-term survival (Moonesinghe et al. 2014; Khuri et al. 2005; Toner and Hamilton 2013). It therefore follows, that the outcome of surgery should not be defined entirely by short-term survivorship. Longer-term and additional patient-centred outcomes such as quality of life or disability-free survival are increasingly viewed as important measures of perioperative ‘success’ by both clinicians and patients, but there is comparatively little high quality data on these outcomes (Boney et al. 2016; Myles et al. 2016). This is particularly important given the evolving demographics of the surgical population (increasing age and multi-morbidity) and the increased number of surgical options available for previously untreatable conditions. There is also a recognition that not all interventions are in the best interests of patients, and sometimes less invasive approaches may lead to better outcomes and fewer adverse events (Skou et al. 2015; Kise et al. 2016). Thus, understanding the full implications of a surgical intervention is critical to supporting high quality patient care and shared decision making.

Beyond understanding these issues more clearly, there is also a need to measure and improve structures and processes associated with perioperative care, so that outcomes may also improve. Numerous national audit and quality improvement initiatives are already operational in the UK, ranging from short-term evaluations of specific clinical areas (e.g. National Confidential Enquiry into Patient Outcome and Death (NCEPOD)) to longer-term continuous national registries and audits (e.g. the National Joint Registry or the National Emergency Laparotomy Audit (NELA)). While these audits are important for national monitoring and quality assurance, the use of data for improvement by clinical teams is variable, despite basic reports of local data being available for review immediately upon electronic data entry. It is common that local teams wait for national reports to be published to take action on their local performance, and the lag time between data collection and publication of national results can therefore be a barrier to quality improvement (QI). Thus, the use of data to drive local QI activity in these different programmes varies considerably, both because of the variation in the methodology of the programme (e.g. ACS-NSQIP provides only annual reporting) and because of variation in the capacity and capability of clinical teams to review quality data, even if available to them prospectively and continuously, driven by culture, resource availability and clinician engagement (Allwood 2014).

The PQIP patient study attempts to address these areas of need through a comprehensive national programme of data collection and feedback, incorporating a multi-level complex intervention aimed at supporting the use of data for improvement at local level. We describe here the protocol for the data collection and feedback. The complex intervention will be described and evaluated in separate papers.

## Methods

### Main study design

Prospective cohort study of patients undergoing major surgery in NHS hospitals. Observational study at patient level; interventional action research study using time-series analysis at hospital level.

### Aims

1. To measure processes of care and outcome in patients undergoing major surgery in the UK NHS.
2. To create a national resource for the collection, management and analysis of high-quality perioperative data, to support collaborative research and efficient study design.
3. To implement and evaluate a complex intervention aiming to enhance the use of data for improvement by clinical teams

### Objectives

1. To comprehensively measure and report structures, processes and risk-adjusted complications, patient-reported outcome and mortality rates after major surgery
2. To evaluate the relationships between structures, processes and outcomes after major surgery
3. To develop bespoke, regularly updated and non-proprietary risk models for the purposes of case-mix adjustment and perioperative risk prediction for different outcomes
4. To support local quality improvement through feedback of data to clinicians and managers using near-real-time feedback and regular comprehensive reporting.
5. To develop and evaluate the effectiveness of a theoretically underpinned complex intervention involving novel methods of data feedback, analysis and support mechanisms to aid local improvement initiatives.
6. To support the development and delivery of collaborative nested studies.

### Research questions

1. What is the level of compliance with evidence-based structures and processes for patients undergoing major surgery?
2. What are the rates of complications, failure to rescue (FTR), short- and long- term mortality in patients undergoing major planned surgery in NHS hospitals?
3. How do the rates of adverse outcomes vary over time and between institutions?
4. Are there structural or process related predictors of these adverse outcomes?
5. What is the relationship between different measures of postoperative outcomes (e.g. different measures of morbidity; short and longer-term outcomes, health-related quality of life and disability)?
6. What is the validity and predictive performance of previously published risk prediction models for different perioperative outcomes in the PQIP population?
7. Can existing risk models be improved, or more parsimonious versions developed to support temporally responsive risk adjustment and clinically useful risk evaluation?
8. How representative is the sample of patients recruited to PQIP of the total cohort of patients undergoing potentially eligible procedures in PQIP hospitals?
9. Is there an outcome benefit to hospital and/or patient involvement in PQIP which can be differentiated from secular variation?
10. Can quality of care be improved through a theoretically underpinned complex intervention supporting the use of data for improvement at local level, and do patient outcomes also improve?

### Ethics

The study has been approved by the Health Research Authority (HRA) as a research study, following review at the South-East Coast—Surrey Research Ethics Committee (REC reference: 16/LO/1827; IRAS project ID: 215,928). All hospitals provide Caldicott Guardian approval in addition to confirmation of capacity and capability in accordance with usual HRA requirements.

### Participants

All NHS hospitals which undertake any of the included procedures are eligible to participate. Hospitals are approached via the Royal College of Anaesthetists and its Health Services Research Centre, and via the National Institute for Health Research’s portfolio system. Patients aged 18 years or older undergoing planned major surgery (as defined by a list of index procedures requiring inpatient stay) will be eligible for recruitment and patient consent will be sought pre-operatively. Patient consent is required as the study seeks additional data to that collected as part of routine care (specifically the patient-reported outcome data). The consenting process is undertaken by staff locally trained and approved. The participant information provides detail on how data are collected, the request for patient reported data, and the linkage of hospital data with external databases (see below, dataset).

Hospitals may choose to recruit patients from all specialties or specify particular specialties where they wish to focus at local level. Each hospital is offered the opportunity to either approach all eligible patients for consent, or all patients within a particular specialty or plan to recruit between one and five patients per week, approaching patients for written consent based on a random sampling strategy. This will involve an eight-day rolling sampling cycle (i.e. for week one the first five patients starting on Monday morning, followed by the first five patients starting from Tuesday in week two, et cetera). If any of the first five patients approached decline to consent, then consecutive patients will be approached for consent until the target recruitment number has been achieved. Patients can choose to be withdrawn from the study at any time.

#### Sample size

Target recruitment is 70,000 patients. It is expected that this will take at least 5 years to achieve. The long observation period will facilitate the study of how data collection and feedback impacts upon patient outcomes over time.

### Dataset

The dataset has been informed using the best available evidence. The primary outcome is the presence of any POMS-defined morbidity on postoperative day seven. Secondary outcomes will include: POMS-defined major morbidity (Wong et al. 2017); complications graded according to the Clavien-Dindo classification; failure to rescue (FTR); resource utilisation (critical care admission (planned/unplanned); critical care length of stay, hospital length of stay, hospital readmission within 30 days of index procedure); mortality at 90 days; days alive and out of hospital (Moonesinghe et al. 2017)censored at 30 days, 60 days, 90 days and one year postoperatively; disability-free survival at one year; change in patient reported health related quality of life (HRQOL) between baseline and 6 and 12 months after surgery; the responses to the “Ask2Questions” brief questionnaire about complex pain (Faculty of Pain Medicine, London, UK 2022). The choice of outcome measures has been based on formal validation studies (Grocott et al. 2007; Dawson et al. 2001; Shulman et al. 2015)and expert consensus (Clavien et al. 2009; Hutchings et al. 2012; Hutchings et al. 2013; Hutchings et al. 2014; Neuburger et al. 2011; Neuburger et al. 2012; Neuburger et al. 2013; Neuburger et al. 2013). To calculate FTR, we will follow previously described methodology, but using the presence of a Clavien-Dindo grade II or above complication as our definition of postoperative complications (Silber et al. 1992; Silber et al. 2007). Given the changes in how perioperative care is coordinated (e.g. enhanced recovery programmes aiming for early discharge) we will also use different endpoints for mortality to compare and contrast different approaches to how FTR could be measured in the modern era – for example, inpatient death vs. 30-day mortality vs. 60-day mortality). Candidate variables for risk adjustment have been selected from the results of a systematic review, and subsequent original research (Moonesinghe et al. 2013; Protopapa et al. 2014; Canet et al. 2010). These include: physiological measurements (e.g. blood pressure); long-term conditions and their control (e.g. diabetes and HbA1C and heart failure / New Your Heart Association grade) and laboratory results (e.g. haematological and biochemistry assays), as well as more novel measures such as deprivation index. Process measures have been informed by systematic review (Chazapis et al. 2018) and Delphi consensus process. An expert panel (the Clinical Reference Group) of stakeholders from different medical and surgical specialties provided further input to refine the long-list of potential measures. These include measures of staffing (level of seniority of surgeons and anaesthetists involved in care delivery), intraoperative anaesthesia and surgical practice, and enhanced recovery metrics before and after surgery. In addition to prospectively collected data, we will also link patient-level data with NHS Digital Hospital Episode Statistics and the Office of National Statistics mortality register. We will also consider patient-level linkage with other registries such as the National Cancer Registry, the Intensive Care National Audit and Research Centre’s Case-Mix Programme and relevant National Clinical Audits, in order to provide a comprehensive dataset at lowest local data collection burden.

#### Study flow

Patients are approached for participation either by mail, at the preoperative assessment clinic or on the day of surgery. A minimum of 1 h is allowed for patients to consider the information on the participant information sheet before they are asked whether they wish to consent. At the time of consent, patients are asked to indicate whether they wish their longer-term follow-up to be by email or telephone. Following consent, the patient is asked to complete baseline demographic and HRQOL data. On the day of surgery, clinical teams or research staff complete preoperative, intraoperative and recovery room data. Further objective data capture occurs on day 2 or 3 (the Drinking Eating and Mobilising process measure (Levy et al. 2016)), day 7 (the Post-Operative Morbidity Survey (POMS) (Grocott et al. 2007)) and at hospital discharge (the Clavien-Dindo complications grading scale (Clavien et al. 2009; Dindo et al. 2004), and information about the length of stay and post-discharge destination of the patient. In addition, patients are asked to complete a patient satisfaction with anaesthesia questionnaire (Bauer et al. 2001; Walker et al. 2016)within 24 h of surgery, a quality of recovery questionnaire at baseline (at the time of consenting to participate in PQIP) and postoperative day 3 (Stark et al. 2013; Chazapis et al. 2016), and longer-term HRQOL and disability-assessment questionnaires at baseline, 6 and 12 months after surgery (Dawson et al. 2001; Shulman et al. 2015).

#### Loss to follow-up

We are conscious that with a large pragmatic study of this nature without substantial funding but with multiple follow-up points and hospitals participating, that there is the potential for significant loss to follow-up. We have considered this as follows:

Inpatient morbidity and complication follow-up rates are predicted to be high, as they are collected in-hospital. We have created logic checks within our online data entry tool to enable us to differentiate between missing data and negative responses. Hospitals with large amounts of missing data will be contacted and offered support.

Mortality follow-up rates are likely to be high, as availability of the information does not depend on patients being contactable or willing to respond to a questionnaire (through linkage with NHS databases). There will be a small number of patients for whom data linkage is not possible (for example if they have left the NHS) but we will receive information about this type of loss to follow-up from NHS Digital.

For longer-term patient-reported outcomes, we will take measures to maximise response rates, for example through multiple reminder letters & phone calls. Additionally, and where required and appropriate, we will conduct sensitivity analyses to understand if there are any important differences between responders and non-responders and to assess for potential biases.

#### Co-enrolment

There is no barrier to PQIP patients being co-enrolled into other studies. We actively seek collaboration with other researchers to facilitate co-enrolment with other studies while paying attention to the need to minimise redundant data collection and data collection fatigue of patients and investigators.

#### Data sharing

Local data can be exported by approved local investigators at each hospital at any time. We will invite PQIP collaborators to apply for multi-centre data to undertake their own secondary analyses which are outside our initial analysis plans. These applications will be reviewed by the PQIP project team and assistance offered for analysis and interpretation, subject to capacity. Applications for access to PQIP data by non-PQIP collaborators will be considered when the study and the planned analyses are completed. Fully anonymised datasets will be made available on data sharing resources once the study and all follow-up is complete.

## Analysis plan

### Descriptive statistics (RQ1, RQ2)

Descriptive statistics will be used to describe the basic demographics of participants. We will report process measures of engagement with PQIP (case-ascertainment rates, data completion) and compliance with processes of care at local and national level. (RQ1) Both unadjusted and adjusted outcomes will be reported (see inferential statistics for details of risk adjustment). (RQ2) All hospital level data will be presented anonymously.

### Missing data

For each statistical analysis, we will document the number and rate of missing observations on all variables involved. For each statistical model, we will assess the likely process that lead to missing observations, and whether data are likely to be missing at random (MAR) or missing not at random (MNAR). This assessment will inform a decision about the appropriate method of analysis. For example, multiple imputation or imputation of normal values may be considered for predictors in statistical models, depending on the likely process of missingness. Patients who are missing outcome data will be omitted from the relevant analysis; sensitivity analyses will be conducted to evaluate differences between patients with and without missing outcome data.

### Temporal and between-hospital variation in outcomes (RQ3)

We will assess the variation over time and between hospitals on all primary and secondary outcome measures. Risk-adjustment will be based on logistic regression or other regression models, as appropriate for each outcome measure. Both patient-level and operation-level predictors will be included, such as age, gender, operation type and the constituent variables of previously published and validated risk adjustment models such as the Portsmouth Physiological and Operative Score for the enumeration of morbidity and mortality (P-POSSUM) and the Surgical Outcome Risk Tool (SORT) (Prytherch et al. 1998; Protopapa et al. 2014).

### Association between outcomes and hospital structures and processes of care (RQ4)

To describe the relationship between outcomes and hospital structures and processes, we will use mixed-effects models, employing random coefficients to assess the variation between hospitals. Patient-level covariates will be included as appropriate to distinguish the effect of case-mix from the effect of hospital characteristics on outcomes.

### Relationship between different postoperative outcomes (RQ5)

We will evaluate the association between different outcome measures which purport to assess similar constructs (e.g. short-term morbidity) and between outcomes which may be associated with each other. As an example for the latter, the relationship between short-term complications and long-term survival has been demonstrated previously (Moonesinghe et al. 2014; Khuri et al. 2005; Toner and Hamilton 2013); however, there are fewer data evaluating the relationship between a complicated postoperative course and health-related quality of life or disability free survival. We will use odds ratios, Pearson correlations and statistical measures of agreement as appropriate.

### Evaluation of existing risk prediction models (RQ 6)

We will evaluate discrimination (using the area under receiver-operator-characteristics curves) and calibration (using Hosmer–Lemeshow or Pearson correlation statistics) of known risk prediction models including the P-POSSUM, SORT and Surgical Risk Scale.

### Developing new models for the prediction of risk (RQ 7)

We will develop and internally validate new models for the prediction of risk, using logistic or other regression models as appropriate. Penalised regression models will be considered to reduce the risk of overfitting. These analyses may lead to a modification of the dataset with the aim of reducing data collection burden, if we find parsimonious models that are able to do without some variables previously considered important for risk prediction.

### Evaluating sample validity (RQ8)

One of the objectives of PQIP is to measure and report processes and outcomes from surgery for the purposes of quality improvement. While it is widely accepted that data from a non-random sample of patients within an institution may provide important information about reliability of processes and systems (Peden and Moonesinghe 2016), sampling of patient outcomes should be statistically and conceptually robust so as not to provide biased estimates. Clinical trials and cohort studies which have specific inclusion and exclusion criteria, or which require patient consent, can be criticised as non-representative (and therefore the findings are of limited generalisability) (Deaton and Cartwright 2018). A potential solution to this is to use administrative data to evaluate outcomes; however, in the UK, such data are limited to mortality, hospital readmission and length of stay.

We will address these issues through an evaluation of our sampling strategy using administrative data from Hospital Episode Statistics (HES). For included procedures, an anonymised extract of HES data will be requested from NHS Digital for each hospital. This will enable us to conduct sensitivity analyses comparing patient characteristics (for example age, comorbidity, socioeconomic status using the Index of Multiple Deprivation) and outcomes (mortality, hospital readmission, length of hospital stay) between patients included in PQIP and those who are not. We will do this at individual hospital level and at aggregate (national) level.

### Evaluating impact of PQIP (RQ9)

We will compare risk-adjusted patient outcomes which are available from administrative data (e.g. mortality, length of stay, readmission to hospital, ‘days alive and out of hospital’ at 30 and 90 days and one year after surgery (Moonesinghe et al. 2019)) in hospitals and patients who are enrolled in PQIP and those who are not. Temporal trends will be analysed using a difference in differences approach. In order to avoid bias associated with different hospital types which may affect patient outcomes irrespective of involvement with PQIP, we will apply coarsened exact matching (Iacus et al. 2012; Bonfrer et al. 2018), matching PQIP and non-PQIP hospitals using pooled data from organisational surveys conducted by the Health Services Research Centre for PQIP and other studies (e.g. SNAP2-EPICCS (Moonesinghe et al. 2017)and the National Emergency Laparotomy Audit (National 2014)) as well as open-access external sources such as NHS England and Department of Health datasets.

## Embedded further research

### PQIP process evaluation (RQ10)

One of the core aims of PQIP is to enhance the use of data for improvement by clinical teams, and in so doing, improve processes and outcomes for patients. PQIP may be considered as a complex intervention, which has been developed based on two theoretical frameworks (Normalisation Process Theory and the Theoretical Domains Framework). The intervention involves every part of the measurement, audit and feedback cycle and beyond, and includes collaborative events, regular reporting, dashboards of key measures and the use of social media and educational media to disseminate knowledge and learning. We are evaluating the effectiveness and processes of implementation of PQIP (RQ9) using both qualitative and quantitative methods. Quantitative methods will include the temporal analysis described to address RQs 3 and 9, and longitudinal survey work which will evaluate engagement with the use of PQIP data. The qualitative research includes multi-sited ethnography, using interviews and observations with frontline staff across PQIP and non-PQIP sites and PQIP project team members. The protocol for the qualitative research has been published separately (Wagstaff et al. 2019).

### Nested studies and secondary use of PQIP data

The multi-centre nature of PQIP provides an opportunity to develop and conduct multiple further studies in sub-groups of patients or hospitals, to pilot and evaluate new interventions, and then consider wider implementation. Such interventions may be aimed at improving processes and outcomes of care for patients (core aim 1 of PQIP) or improving the use of data for improvement (core aim 2 of PQIP). Two examples are briefly outlined below.

### pomVLAD

Post-Operative Morbidity reporting using Variable Life Adjusted Displays (pomVLAD), is a nested study funded by the Health Foundation which will develop, implement and evaluate the effectiveness of a near real-time reporting system for risk-adjusted morbidity, mortality and FTR rates. Originally developed to monitor observed versus expected mortality in cardiac surgery, VLAD provides a graphical display of risk-adjusted outcome data over time (Pagel et al. 2013). We plan to develop a recommendation bundle which will be paired with the VLAD display. The enhanced recovery bundle and VLAD dashboard will be trialled in ten early adopter hospitals and evaluated using a difference-in-differences analysis to evaluate its effectiveness and feasibility (Etzioni et al. 2015). If this pilot work achieves its feasibility aims, then there will be potential for wider roll-out of pomVLAD to support quality improvement activity at local level.

### ERAS plus

The ERASPlus initiative (erasplus.co.uk) is an expansion of a quality improvement programme aimed at reducing postoperative pulmonary complications. It was initially implemented, evaluated and found to be clinically effective in a single centre (Moore et al. 2017) and is now being rolled out throughout the Greater Manchester area. The intervention consists of a bundle of care supported by technological innovation (an app for patients to use) including exercise advice and training and targeted interventions to improve pulmonary function and reduce the risk of complications. The expanded ERASPlus initiative is also being funded by the Health Foundation and is working with PQIP to combine data collection efforts to reduce burden on local teams; additionally, this will facilitate comparisons with the non-ERAS + population of PQIP patients.

The PQIP project team will continue to consider requests from external clinicians and researchers collaborate and modify the PQIP dataset to maximise its usefulness for QI or research. After our planned analyses have been completed, a process will be established to enable external clinicians and researchers to apply for access to an anonymised dataset to explore research hypotheses.

## Data management and linkage

All investigators and study site staff comply with the requirements of the Data Protection Act 1998 with regards to the collection, storage, processing and disclosure of personal information and will uphold the Act’s core principles. Patient-level data is entered by local investigators into a secure, electronic, web-based database. This is hosted on servers managed by UK Fast on behalf of the RCoA. Local investigators have access to their own full datasets. An anonymised dataset will be used by the PQIP Project Team for analysis. In this dataset: NHS number is replaced by a unique study patient identifier; date of birth is converted to age on date of surgery; postcode is converted to primary care trust (PCT), strategic health authority (SHA) of residence and the ONS Lower Super Output Area to allow the allocation of the Index of Multiple Deprivation.

The minimum amount of patient identifiable data (including PQIP unique identifier, NHS number, date of birth, gender and postcode) will be extracted from the study database by the PQIP Project Team to allow data linkage to ONS mortality data and Hospital Episode Statistics data from NHS Digital, so that we may track post-discharge outcomes (e.g. hospital readmission) and long-term survival.

## Patient and public involvement

The PQIP patient study addresses areas prioritised by four James Lind Alliance Priority Setting Partnerships (JLA-PSPs): anaesthesia/perioperative care, intensive care, dementia & pressure ulcers. We received detailed structured feedback on our protocol from members of the PCPIE group at the National Institute for Academic Anaesthesia's Health Services Research Centre (NIAA-HSRC). Our project team has two lay members, as does our clinical reference group which provides support and advice on request. Patient representatives are full members of the study team and as such, are invited to comment on all aspects of continuing study development and implementation.

## Impact and dissemination

A report of key findings is produced annually. Quarterly study reports are disseminated to participating sites and published on the study web site. Research resulting from the study will be disseminated by presentations and publications in open-access peer-reviewed journals. Wider dissemination to various stakeholders, including the public, will be achieved via social media, podcasts, short videos, press releases, and written and electronic communications.

## Discussion

PQIP has now been running for 4 years, and was paused for approximately one year during the Covid pandemic, both because elective surgery activity reduced substantially, and because clinical and research teams were diverted to support the emergency response. Review of data collected to that point and discussion with clinical teams has found that the random sampling approach was largely infeasible at hospital level. Therefore, a convenience sampling approach has been adopted by most sites. This further underlines the importance of our planned analyses to evaluate risk of bias in our patient selection and our results.

This also highlighted another challenge with our approach: the direct cost of participation in the study. Hospitals are incentivised to take part because the PQIP patient study is registered on the National Institute for Health Research’s portfolio of approved studies: therefore, sites are financially rewarded for recruiting patients. However, the costs of data collection and entry must be covered locally, and this is a significant challenge, which has limited both site participation and the number of patients recruited per site. Nonetheless, over 126 hospitals have taken part in the PQIP patient study so far, and over 35,000 patients have been recruited.

PQIP has also successfully collaborated with another research team to facilitate an NIHR funded randomised controlled trial to use the PQIP data collection platform and co-recruit into both studies (https://www.journalslibrary.nihr.ac.uk/programmes/hta/NIHR130573/#/) This approach to efficient trial design should bring benefit to patients, clinicians, researchers and provide excellent value for money.

## Conclusion

The PQIP Patient study is the UK’s first national effort to report and improve patient outcomes from major surgery other than mortality and length of stay. A number of research questions will be answered alongside the main purpose of quality improvement in perioperative patient care. The programme is notable in that it is being formally evaluated while in progress.

## Data Availability

Not applicable.
