# Correction: The Perioperative Quality Improvement Programme (PQIP patient study): protocol for a UK multicentre, prospective cohort study to measure quality of care and outcomes after major surgery

**DOI:** 10.1186/s13741-025-00610-z

**Published:** 2025-12-03

**Authors:** S. Ramani Moonesinghe, Dermot McGuckin, Peter Martin, James Bedford, Duncan Wagstaff, David Gilhooly, Cristel Santos, Jonathan Wilson, Jenny Dorey, Irene Leeman, Helena Smith, Cecilia Vindrola‑Padros, Kylie Edwards, Georgina Singleton, Michael Swart, Rachel Baumber, Arun Sahni, Samantha Warnakulasuriya, Ravi Vohra, Helen Ellicott, Anne‑Marie Bougeard, Maria Chazapis, Aleksandra Ignacka, Martin Cripps, Alexandra Brent, Sharon Drake, James Goodwin, Dorian Martinez, Karen Williams, Pritam Singh, Matthew Bedford, Abigail E. Vallance, Katie Samuel, Jose Lourtie, Dominic Olive, Christine Taylor, Olga Tucker, Giuseppe Aresu, Andrew Swift, Naomi Fulop, Mike Grocott

**Affiliations:** 1 Centre for Perioperative Medicine, Research Department for Targeted Intervention, UCL, London, UK; 2 Health Services Research Centre, Royal College of Anaesthetists, London, UK; 3 Department of Anaesthesia and Perioperative Medicine, University College London Hospitals NHS Foundation Trust, London, UK; 4 Department for Applied Health Research, UCL, London, UK; 5 Department of Anaesthesia, York Teaching Hospitals NHS Foundation Trust, York, UK; 6 Royal College of Anaesthetists, London, UK; 7 Department of Anaesthesia, Royal National Orthopaedic Hospital, Stanmore, UK; 8 Department of Anaesthesia, Torbay Hospital, Torquay, UK; 9 Department of Upper GI Surgery, Nottingham University Hospitals, Nottingham, UK; 10 NetSolving Limited, Croydon, UK; 11 Trent Oesophago-Gastric Unit, Nottingham University Hospitals NHS Trust, Nottingham, UK; 12 Department of Colorectal Surgery, Birmingham Heartlands Hospital, Birmingham, UK; 13 Bristol Medical School, University of Bristol, Bristol, UK; 14 Department of Anaesthesia, North Bristol NHS Foundation Trust, Bristol, UK; 15 Department of Upper Gastrointestinal Surgery, Heartlands Hospital, Birmingham, UK; 16 Department of Cardiothoracic Surgery, Papworth Hospital, Cambridge, UK; 17 Aintree Hospital, Liverpool, UK; 18 Division of Critical Care, University of Southampton, Southampton, UK

**Correction** **:** **Perioper Med 11, 37 (2022)**

**https://doi.org/10.1186/s13741-022–00262-3**

Following the publication of the original article (Moonesinghe et al. 2022), the authors identified an error in the Competing interests section. The section should read:

**Competing interests**

Mike Grocott is the Editor-in-Chief of the Perioperative Medicine.

**The old statement of the Competing interests:** None.

The Competing interests section has been updated above and the original article (Moonesinghe et al. 2022) has been corrected.
